# Increased growth of colorectal liver metastasis following partial hepatectomy

**DOI:** 10.1007/s10585-013-9572-y

**Published:** 2013-02-06

**Authors:** P. Krause, H. Flikweert, M. Monin, A. Seif Amir Hosseini, G. Helms, G. Cantanhede, B. M. Ghadimi, S. Koenig

**Affiliations:** 1 Department of General and Visceral Surgery, University Medical Centre, Georg-August-University Goettingen, Robert-Koch-Strasse 40, 37075 Goettingen, Germany; 2 Department of Diagnostic Radiology, University Medical Centre, Georg-August-University Goettingen, Goettingen, Germany; 3 Department of Cognitive Neurology, MR-Research in Neurology and Psychiatry, University Medical Centre, Georg-August-University Goettingen, Goettingen, Germany

**Keywords:** Colorectal metastases, CC531, WAG/Rij rat model, Liver regeneration, Partial hepatectomy, Magnetic resonance imaging

## Abstract

Nearly 50 % of colorectal cancer (CRC) patients develop liver metastases with liver resection being the only option to cure patients. Residual micrometastases or circulating tumor cells are considered a cause of tumor relapse. This work investigates the influence of partial hepatectomy (PH) on the growth and molecular composition of CRC liver metastasis in a syngeneic rat model. One million CC531 colorectal tumor cells were implanted via the portal vein in WAG/Rij rats followed by a 30 % PH a day later. Control groups either received tumor cells followed by a sham-operation or were injected with a buffer solution followed by PH. Animals were examined with magnetic resonance imaging (MRI) and liver tissues were processed for immunolabeling and PCR analysis. One-third PH was associated with an almost threefold increase in relative tumor mass (MRI volumetry: 2.8-fold and transcript levels of CD44: 2.3-fold). Expression of molecular markers for invasiveness and aggressiveness (CD49f, CXCR4, Axin2 and c-met) was increased following PH, however with no significant differences when referring to the relative expression levels (relating to tumor mass). Liver metastases demonstrated a significantly higher proliferation rate (Ki67) 2 weeks following PH and cell divisions also increased in the surrounding liver tissue. Following PH, the stimulated growth of metastases clearly exceeded the compensation in liver volume with long-lasting proliferative effects. However, the distinct tumor composition was not influenced by liver regeneration. Future investigations should focus on the inhibition of cell cycle (i.e. systemic therapy strategies, irradiation) to hinder liver regeneration and therefore restrain tumor growth.

## Introduction

The liver is one of few organs with the capability of regenerating fully in capacity and function. Currently, partial hepatectomy (PH) is an effective means of curing patients from various lesions of this organ within the clinical setting of a multimodal approach [1]. Furthermore, surgery has unavoidably been drawn into having to understand the process of liver regeneration. For over 60 years, PH has been performed to resect liver primaries such as hepatocellular carcinoma or liver metastasis and with time, metastatic disease became the most frequent indication for partial liver resection [2].

Colorectal cancer (CRC) is the third most common cancer in the Western world [3], and the second leading cause of death in both genders; nearly half of all affected patients develop liver metastasis [4]. The prevalence is around 15–30 % in newly diagnosed cases [5], and an additional 25 % of cases eventually develop metastasis within 2 years. Despite the resection of tumor-affected lobes, the 5-year survival still borders on 30 % [6].

Following resection of the primary, systemic minimal residual disease can remain asymptomatic for periods that can last years or even decades. This is due to the presence of disseminated tumor cells in different organs. In particular, several cancers including melanoma, breast, prostate, and colorectal carcinoma undergo dormant periods before metastatic recurrence [7]. This metastatic rebirth may be initiated by an imbalance of various factors or mechanisms contributing to cellular changes shifting apoptosis towards proliferation (e.g. release of proliferative factors by the micro-environment), by an increase in angiogenesis or evasion of the immune system [8]. These three conditions have to be considered when tumors are re-initiated as metastases in secondary sites such as the liver. However, metastatic relapses following curative resection of hepatic metastases may principally originate from residual and undetectable micrometastasis in the remaining liver parenchyma or circulating single tumor cells rather than dormant tumor cells. In this context, PH may trigger a myriad of proliferative signals that provoke the proliferation of residual or circulating cancer cells, which are consequently stimulated into fast and novel tumor formation. In humans, metastatic disease (intrahepatic or extrahepatic) usually re-occurs within 36 months following hepatic R0 resection. Moreover, recurrence-free survival in patients following PH is 10.6 months in former bilobar and 16.1 months in unilobar manifestations of CRC liver metastasis [9]. Despite these sobering observations, even in advanced stages of CRC disease and various concepts of systemic therapy, liver resection remains quintessential to the successful treatment of patients and we will certainly have to look into new strategies as to how to modify (reduce) the regenerative response following PH at some stage in the future to improve patient prognosis.

Our workgroup reported on a complex culture system capable of mimicking the micromilieu of the regenerating liver in vitro [10]. We incubated primary mature rat hepatocytes in this liver-specific growth medium (supplemented with mitogens and conditioned media derived from hepatocytes and stromal cell culture supernatants). These adult liver cells were subsequently stimulated into proliferation. Aiming to investigate the underlying mechanisms and factors by which liver regeneration may trigger the growth of tumor cells, we employed the same culture system and conditions. And indeed, we observed that CRC cells (rat cell line CC531) were stimulated into rapid expansion and significantly higher proliferation in this growth medium than that noted in control media (RPMI plus 2 % FCS) (unpublished data). With these encouraging results in mind, we then investigated the growth of tumor cells in a preclinical rat animal model of orthotopic CRC liver metastasis. We therefore implemented and modified an experimental rat model, in which CC531 tumor cells were implanted into syngeneic WAG/Rij rats [11]. Referring to frequent mutations in human CRC, the rat cell line CC531 is also known to display a prototypic beta-catenin (Ctnnb1) mutation (Thr(41)Ile) as well as a ki-ras (G12D) mutation, providing unambiguous evidence of the constitutive activation of these pathways [12]. The injection of these tumor cells via the portal vein leads to the reproducible formation of liver metastases and therefore reflects the human, weakly immunogenic, tumor-host relationship well and thus provides an excellent opportunity to study tumor cell growth under the influence of PH.

In the present study, we hypothesize that liver regeneration triggers a course of molecular signaling and the subsequent activation of pathways which accentuate the proliferation of tumor cells and finally lead to the increased formation of metastasis in the liver parenchyma. We investigated the metastatic ability and behavior (for example: spatial growth and expansion, expression of molecular features as well as markers of invasiveness and proliferative effects both on the metastases as well as on the surrounding liver tissue). We were particularly interested in quantifying the tumor burden and identifying molecular pathways which mediate and/or enhance the tumor spread following PH.

## Materials and methods

### Animals and reagents

Male WAG/Rij rats (weight 160–200 g) were purchased from Charles River (Sulzfeld, Germany). Animals were kept on 12-hour day/night rhythm and fed with a standard rat diet (ssniff, Soest, Germany). All animal breeding, care and experimentation procedures were in accordance with the German national and regional legislation on animal protection.

Unless specified otherwise, all chemicals and reagents were supplied by Sigma-Aldrich (Deisenhofen, Germany). Fetal calf serum (FCS) was purchased from PAN (Aidenbach, Germany) and trypsin 10-fold was supplied by PAA (Pasching, Austria). Primary antibodies were purchased and used as illustrated in Table 1. Secondary fluorescence labeling antibodies (Alexa Fluor) were obtained from Molecular Probes (Goettingen, Germany). Tumor cells CC531 were supplied by CLS (Eppelheim, Germany) and cultured in 75 cm^2^ cell flasks (Sarstedt, Nuembrecht, Germany).

**Table 1:** Antibodies for co-localization studies

Antibody	Species	Manufacturer	Dilution
CD44	Mouse monoclonal IgG2a, clone ox-49	BD Pharmingen, Heidelberg, Germany	1:500
Cx32 (gap junction protein)	Rabbit, polyclonal	Sigma, Saint Louis, MO, USA	1:5,000
Frizzled (wnt-receptor)	Goat, polyclonal	R & D Systems, Wiesbaden, Germany	1:50
CD49f	Mouse monoclonal IgG1, clone Mab-5A	Acris, Herford, Germany	1:200
Phospho-STAT3 (Tyr705)	Rabbit polyclonal	Cell Signaling, Danvers, MA, USA	1:50
β-Catenin	Rabbit polyclonal	BD Bioscience, Heidelberg, Germany	1:100
Ki67	Rabbit monoclonal, clone SP6	Cell Marque, Rockling, CA, USA	1:500

### Cell culture

CRC cells from the rat cell line CC531 were expanded and stored in frozen aliquots (−70 °C). After thawing, the cells were routinely cultured on 75 cm^2^ culture flasks in RPMI 1640, supplemented with 10 % FCS, 1 % l-glutamine and 1 % penicillin/streptomycin at 37 °C and 5 % CO_2_ in a humidified incubator. After initial seeding, FCS concentration was reduced to 5 % with the first medium change. Tumor cells were passaged once (following 3 days in culture), cultured for a further 4 days, and then trypsinized for subsequent implantation studies. Tumor cells from the same passage were used for all the implantation experiments.

### Animal procedures

In the first step, the stable and reproducible growth of liver metastases as well as phenotypic marker expression was documented following tumor cell implantation. Animals were anesthetized under constant sevofluran inhalation (Sevorane^®^, Abbott, Wiesbaden, Germany) following subcutaneous application of carprofen (Rimadyl^®^, Pfizer, Berlin, Germany) (5 mg/kg body weight). After median laparotomy, the hilum of the liver was exposed to access the portal vein. One million tumor cells in a volume of 250 μl PBS buffer were injected slowly into the portal vein using a 28 G needle. Animals were sacrificed after 3 days, 1 and 2 weeks. Tissue samples from the liver were excised, snap-frozen for PCR-analysis, frozen in 2-methylbutane at −70 °C for fluorescence immunolabeling or processed for paraffin embedding.

After successful establishment of the rat model for CRC liver metastases, we investigated the effects of PH on tumor cell growth. For this purpose, animals were divided into three groups:

Group 1: Tumor cell implantation + 30 % PH (intervention)
Group 2: Tumor cell implantation + mobilization of liver (sham-OP)
Group 3: Injection of buffer solution + PH (control)

Group 1 received an implantation of tumor cells via the portal vein as described above. Twenty-four hours later, re-laparotomy was performed to expose the lateral segment of the left liver lobule, which was removed by central ligation. The laparotomy incision was closed by continuous suture. In Group 2, animals were injected with tumor cells, re-laparotomy being performed 24 h later comprising gentle mobilization of the lateral segment of the left liver lobule. Animals from Group 3 received an injection of buffer solution and underwent PH a day later. Animal experiments were terminated at the latest 2 weeks after tumor cell implantation. Explanted livers were sliced for macroscopic assessment and photographic documentation of the section planes. Thereafter, tissue samples were processed and stored as described above.

### Immunolabeling

Cryostat sections (5 μm) were fixed in ice-cold acetone for 10 min, or paraformaldehyde at room temperature (RT) for 30 min, followed by 70 % ethanol at −20 °C for another 30 min and were finally stored at −80 °C. After rehydration in Tris/HCl buffer (pH 7.6), sections were immunostained for the first antigen (incubation with the first primary antibody anti-CD44 overnight at 4 °C) and further processed with the second primary antibody anti-Connexin 32 (Cx32), anti-CD49f or anti-frizzled. Single staining procedures were performed with anti-signal transducer and activator of transcription 3 (STAT3) and anti-β-catenin. The secondary antibodies Alexa Fluor 555 goat anti-mouse IgG and species-specific Alexa Fluor 488 or 568 IgG (1:400, 1 h at RT) were used for fluorescence visualization. Negative controls were carried out for each antibody by omitting the primary antibody from the protocol. Samples were covered with 50 μl Mowiol 4-88^®^ based mounting medium (Calbiochem, Darmstadt) containing 1.6 μl DAPI/ml and evaluated under a microscope (LEICA DM IRE2, Bensheim, Germany).

Endogenous peroxidase was inactivated on paraffin sections (2 μm) by incubation with 3 % H_2_O_2_ in 100 % methanol for 30 min at RT. The sections were further exposed to the primary antibodies anti-CD44 or anti-Ki67 and the secondary peroxidase labeled antibodies goat anti-mouse or goat anti-rabbit (DakoCytomation K4002, Carpinteria, USA), visualized by 3-amino 9-ethyl-carbazole (AEC) solution and hematoxylin counterstaining.

### Assessment of tumor cell proliferation

Ki67 immunoreaction was evaluated using light microscopy at 200× magnification by two independent observers (review of discordant cases by a supervisor). Reactivity to Ki67 was evaluated by semi-quantitative scoring considering the percentage of positive tumor area and the staining intensity (grading: negative = 0, low = 1, moderate = 2 or strong = 3) [13]. The percentage of positive area was multiplied by the grades leading to an overall scale from 0 (no positive cells) to up to 300 (100 % positive cells). Different regions of interest were defined: non-necrotic areas within the liver metastasis and representative areas in the surrounding liver parenchyma; the latter was further subdivided into the invasion zone (hepatic margin outside the metastases) and hepatic areas distant from the metastases. Seven to 10 fields of view were counted per region of interest.

### Relative quantification using RT-PCR analysis

For mRNA analysis, specimens from the entire liver were collected and defrosted in peqGold TriFast (Peglab, Erlangen, Germany) (1 ml/1 g tissue) and then homogenized using the Ultra-Turrax^®^ (IKA, Bielefeld, Germany). Ten microlitre of the mixture were then filled with 1 ml of pegGold TriFast. Total RNA was isolated according to the manufacturer’s instructions and stored at −80 °C. First strand cDNA was synthesized using an iScript cDNA Synthesis Kit (BioRad, Munich, Germany) with oligo-(dT). PCRs (Sso Fast EvaGreen Supermix, BioRad, Munich) were carried out using an iCycler (BioRad, Munich, Germany) with intron spanning primers (Eurofins MWG, Ebersberg, Germany) (see Table 2), and reactions were normalized to β_2_-microglobuline (β_2_-MG) and hypoxanthine–guanine phosphoribosyltransferase (HPRT) as internal standards. Amplification and expression analysis were performed as recommended by the manufacturer. Quantitative real time PCR was carried out at 30 s 95 °C, 40× (denaturation 5 s 95 °C, annealing/extension 5 s 55–60 °C).

**Table 2:** Primers for expression studies

Gene	Primer (fw/rv)	Annealing temperature (°C)	Accession number	Product size (bp)
CD44	CAGCTTGGGGACTACTTTGC GAGGTCAGCTGCTTCAGTCC	57.8	NM_012924	143
c-met	GGACTTTGTTGGACAGTGACG GATTCCCTCAGTCAGAAACTGG	59.2	NM_031517	104
CD49f	GTGGCCCAAGGAGATTAGC GTTGACGCTGCAGTTGAGG	57.0	NM_053725	233
CXCR4	GATGGTGGTGTTCCAGTTCC CTTGGAGTGTGACAGCTTGG	59.2	NM_022205	106
Axin2	Reference position: 2580 (Qiagen, Hilden, Germany)	60.0	NM_024355	124
HPRT	CTGGTGAAAAGGACCTCTCG ACTTGCCGCTGTCTTTTAGG	58.3	NM_012583	183
β_2_-Microglobulin	GGTGACCGTGATCTTTCTGG TGGGTGGAACTGAGACACG	61.0	NM_012512	145

### Magnetic resonance imaging (MRI)

MRI was used to monitor the growth of liver metastases and to quantify overall tumor burden as well as whole liver volumes 2 weeks following implantation of the CC531 tumor cells. Rats were anesthetized under constant sevofluran inhalation and scanned in a 3T clinical MRI system (Magnetom TIM Trio, Siemens Healthcare, Erlangen, Germany) using an eight-channel receive coil designed for the human wrist (Invivo Corporation, Gainesville, USA). The examination comprised axial T2-weighted turbo-spin-echo (TSE), diffusion-weighted (DW) echo-planar, and T1-weighted gradient-echo MRI. Coronal TSE was used to check for additional extrahepatic metastases. The intrahepatic tumor site was confirmed by loss of the DW signal. The volumetric analysis was performed using OsiriX (Pixmeo, Geneva, Switzerland). Whole liver and tumor margins were manually outlined on each slice of T2w sequences, taking advantage of the high natural contrast between liver (containing iron with high T2 relaxivity and CRC metastases with slower T2 relaxation). Volumes were calculated manually.

### Statistics

Results were expressed as mean ± standard error of the mean (SEM). Differences between two groups were evaluated with the unpaired Student’s *t* test. Analysis of variance (ANOVA) was used to determine statistical differences. A *p* value ≤0.05 was considered significant.

## Results

### Characterization of the CRC liver metastases

**Fig. 1:**
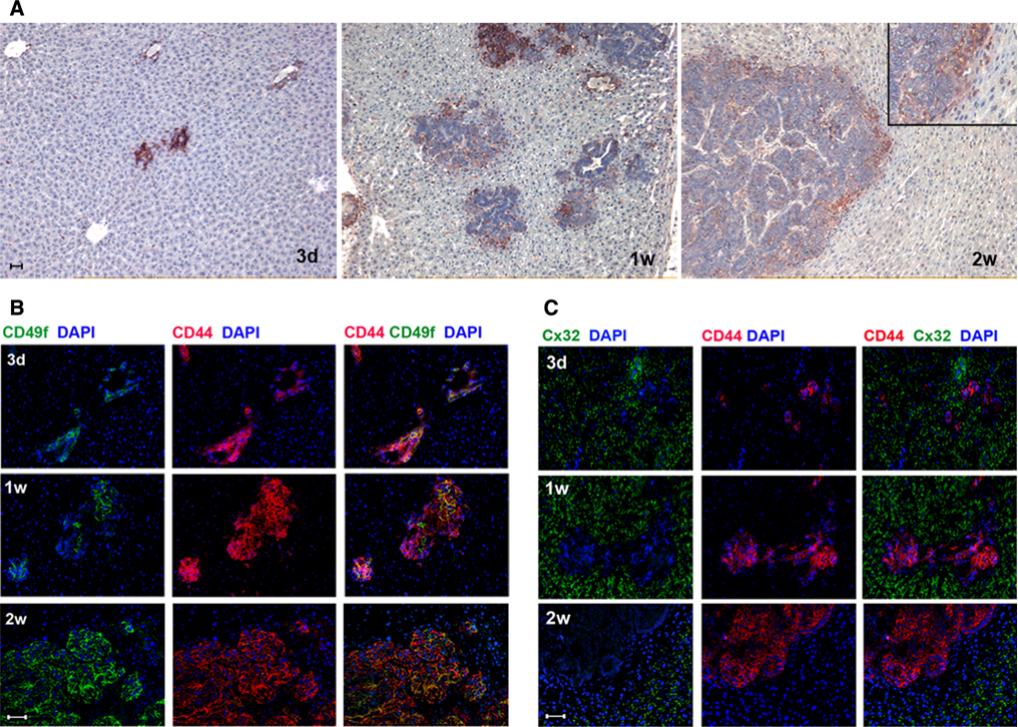
Growth pattern and expression of characteristic tumor markers in expanding CRC liver metastases. Immunohistochemistry and multilayer immunofluorescence stainings for the characterization 3 days (3d), 1 week (1w) and 2 weeks (2w) following implantation of CC531 via the portal vein in WAG/Rij rats. **a** Detection of CD44 to visualize expanding CRC liver metastases which display a pushing growth pattern, which is most evident at 2 weeks (see *insert*). The surrounding hepatocytes are compressed and run in parallel forming a ring-like border with the circumference of the metastasis. **b** Co-localization of CD44 (*red*) as a surrogate parameter to visualize the tumor burden (almost 100 % staining of tumor cells) and the ductular marker CD49f (present in only 70 %) (*green*). **c** Co-localization of CD44 (*red*) and hepatic gap-junction protein Cx32 (*green*). Loss of expression of the latter in hepatocytes found in close proximity to the metastases 2 weeks post PH. FITC (*red*) and TRIC (*green*) fluorescent channels are demonstrated separately and were overlaid in the *last column*. Nuclear counterstaining with DAPI (*blue*), original magnification ×200, *scale bar* = 20 μm. (Color figure online)

**Fig. 2:**
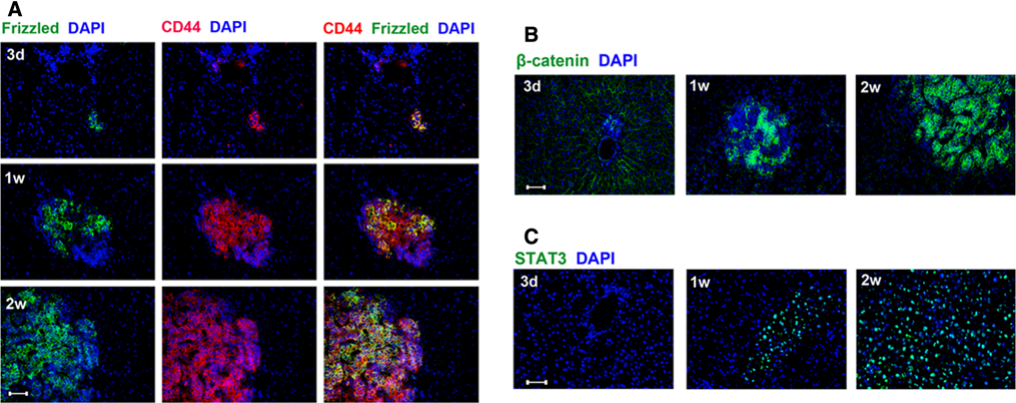
Activation of tumor signaling pathways (wnt and JAK/STAT) in rat CRC liver metastases. **a** Co-localization of CD44 (*red*) and frizzled (*green*). **b** Single staining of β-catenin (*green*). **c** Staining of the transcription factor STAT3 (*green*). Nuclear counterstaining with DAPI (*blue*). Time points as indicated (3d, 3 days; 1w, 1 week; 2w, 2 weeks). Original magnification ×200, *scale bar* = 20 μm. (Color figure online)

On macroscopic assessment, 85–90 % of animals developed liver metastases in any number of liver lobules during the course of the study. However, there was an inhomogeneous spread of tumor burden, with tumors appearing in general larger in size in the right-hand lobes of the liver. On the microscopic level, the kinetics of metastatic growth and phenotypic expression of markers were assessed by immunofluorescence staining 3 days, 1 and 2 weeks following tumor cell implantation. CC531 cells readily implanted into the liver plates and formed metastases gradually increasing in size from small conglomerates (3 days) to moderately sized metastases (1 week) and subsequently large masses growing to confluence at the endpoint of the experiment (2 weeks) (Figs. 1, 2). Liver metastases intensively expressed the hyaluronic acid receptor CD44 (putative marker of ‘stemness’ in CRC) throughout the cytoplasm and cell membranes at all points in time of the experiment (Fig. 1). According to Vermeulen and his workgroup, three different histological growth patterns can be distinguished in human CRC liver metastases: desmoplastic, pushing and replacement [14, 15]. In the rat liver metastases, a ‘pushing’ growth pattern was detected, with a characteristic liver margin in which hepatocytes were ‘pushed’ aside, forming a ring-like border (margin) to the metastases at 2 weeks (Fig. 1a). At the earlier points in time (3 days and 1 week), none of the mentioned growth patterns was at all noticeable, as the tumors at these points were still fairly small and limited. Within the numerous metastases, we found an overall homogeneous expression pattern of CD44 of approximately 100 % staining of all tumor cells. Of note, cytospins from detached tumor cell suspensions (just prior to cell implantation) also demonstrated a high degree of immunoreactivity to CD44 (data not shown). We therefore considered CD44 as a suitable surrogate parameter when tracking tumor cells in vivo. Approx. 70 % of the tumor cells co-expressed the ductular marker CD49f (Fig. 1b). In more detail, those differentiated subclones were arranged in ductular structures and displayed intense staining of CD49f, which was more or less extended over the outer cell membranes.

To visualize the surrounding liver parenchyma, we chose Cx32, a major component of the hepatic gap junction protein, to depict functionally intact hepatocytes (Fig. 1c). At earlier points in time (3 days and 1 week), the hepatocytes in close proximity to the liver metastases appeared unharmed, as they displayed the regular dotted staining pattern. However 2 weeks following tumor cell implantation, the liver metastases were surrounded by a small margin of 2–4 hepatocyte layers, the cells of which expressed Cx32 at a remarkably reduced level if at all. This tumor-liver parenchyma interface corresponded to the ‘pushed’ margin as described above.

The majority of sporadic human CRC display a mutation in the β-catenin dependent wnt-signaling, leading to increased activity of the pathway. With reference to our rat model of CRC liver metastases, we also detected vastly membrane-bound and intense staining of the wnt-receptor frizzled in approximately 50–60 % of tumor cells in forming metastases (Fig. 2a) and a high degree of expression of mostly cytosplasmatic β-catenin in 80–90 % at all investigated points in time (Fig. 2b). Additionally, STAT3, an oncogenic transcription factor, was also found to be constitutively activated in these rat CRC liver metastases, as demonstrated by the near-total (approx. 95 %) positive nuclear staining (Fig. 2c). Owing to technical hindrances, we were unable to co-stain β-catenin or STAT3 with CD44.

### Assessment of tumor burden

MRI was used to locate the metastases and to measure volumes of livers and metastases in the intervention group (PH) and sham-OP group. In summary, MRI confirmed the macroscopic results from animal autopsies: extrahepatic metastasis was detected neither in the lungs nor peritoneal cavity. Furthermore, metastasis was present predominantly in the right liver lobules.

**Fig. 3:**
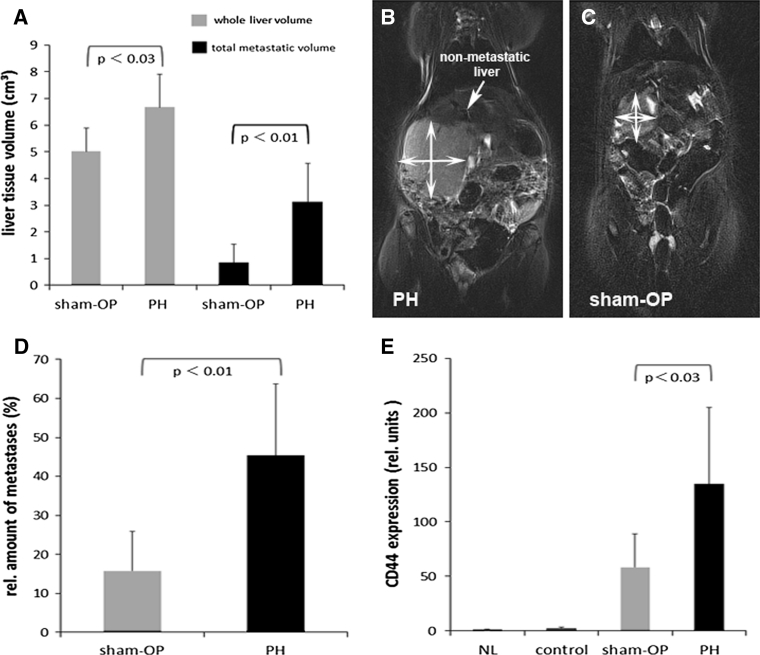
Quantitative assessment of tumor burden and liver volumes 2 weeks following implantation of CC531 into rat livers. **a** MRI volumetric quantification: absolute volumes of whole livers (*grey*) and total metastatic volume (*black*): liver tissue volumes in PH-treated animals increased 1.4 fold in contrast to sham-OP animals. Total metastatic volumes following PH increased up to 3.6-fold in contrast to sham-OP animals. T2-weighted MRI displaying representative coronal sections of rats: prominent and large tumor masses of high contrast in the right liver lobules and only small unaffected (‘non-metastatic’) liver margin after PH (**b**), sham-OP animals revealed limited tumor growth (**c**); the diameters of tumor masses are outlined by *double-headed arrows*. **d** Relative tumor mass (RTM) following PH and sham-OP. Tumor burden increased 2.8-fold after PH. **e** RT-PCR quantification of CD44 gene expression as an surrogate parameter for the metastatic burden revealed a 2.3-fold increase in CD44-transcripts in the PH group when compared to sham-OP; normal livers (NL) and control (buffer + PH) as indicated. Significant levels as indicated. Data are mean ± SEM of 7–10 different animals per group and of three independent experiments (RT-PCR)

Two weeks following tumor cell implantation, the mean total liver volume per animal was 1.3 times greater in the PH group than in the sham-OP group (6.66 vs. 5.02 cm^3^, *p* < 0.03) (Fig. 3a). Representative coronal sections from T2-weighted MRI displayed the intrahepatic high-contrast tumor masses, which were evidently larger in size (as illustrated by their orthogonal diameters) after PH than those from the sham-OP (Fig. 3b, c). More importantly, the growth of metastases following PH exceeded the increase in liver volume. The mean metastatic mass per animal was calculated as 3.1 cm^3^ (PH) compared to 0.85 cm^3^ (sham-OP) (*p* < 0.01), representing a 3.6-fold increase in tumor burden. Relative tumor mass (RTM), defined as (tumor volume/total liver volume) * 100, was determined to be 45 % compared to the sham-OP group, in which the RTM was only 16 % (*p* < 0.01) (Fig. 3d). This corresponds to a 2.8-fold increase.

To confirm these volumetric results, the tumor burden was additionally assessed by detecting CD44 gene expression levels in homogenized rat livers. As seen in Fig. 3e, PH provoked a significantly greater CD44 expression in tumor-bearing livers (*p* < 0.03). The mRNA-level was 2.3 times higher in the intervention group when compared to the sham-OP group. It is worth noting that livers in the control group had levels similar to those exhibited by unharmed, normal liver.

### Molecular composition CRC liver metastases

**Fig. 4:**
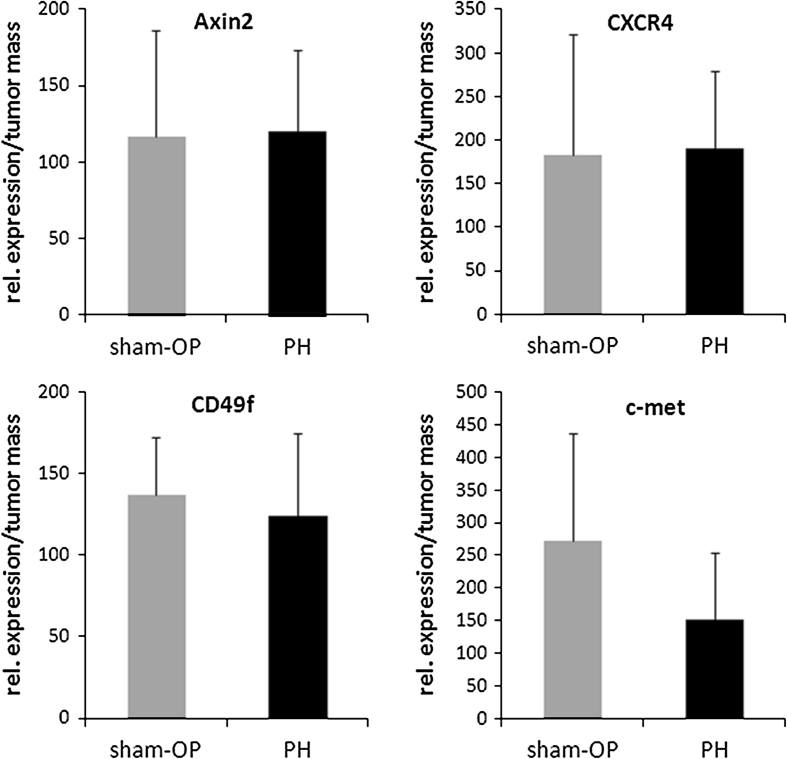
Relative expression of tumor markers correlated to tumor burden (gene levels of homogenized livers were related to the gene expression of CD44). Data are mean ± SEM of 7–10 different animals

RT-PCR revealed changes in the molecular composition of tumor-bearing livers following PH. The transcripts of CD49f, CXCR4 and Axin2 were considerably elevated at the endpoint (*p* < 0.03). The receptor c-met demonstrated a tendency to higher levels of expression in the PH group (data not shown). The relative expression, defined as individual marker gene expression/CD44 expression as a representative of tumor burden and calculated for each individual animal, did not prove to be statistically significant enough to be able to differentiate between the PH and sham-OP group for all 4 markers investigated (Fig. 4).

### Assessment of proliferation in metastases and surrounding liver

**Fig. 5:**
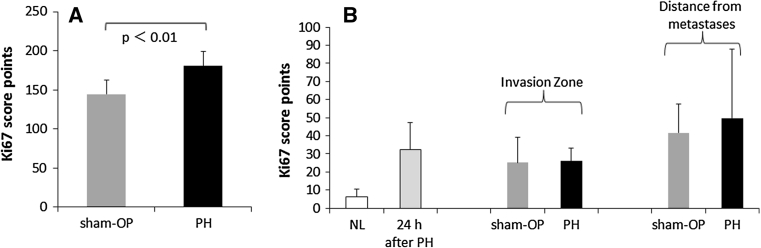
Assessment of cell proliferation by semi-quantitative scoring of Ki67 staining. **a** CRC liver metastases demonstrated high Ki67 scores following PH (vs. sham-OP), *p* < 0.001. **b** Moderate proliferation scores of hepatocytes in the surrounding liver tissue in close proximity to metastases (*invasion zone*) and in distance to the metastases; additional control for comparison = healthy and regenerating liver at 24 h after PH. Data are mean ± SEM of 7–10 different enumerations of the fields of view per region of interest. Significance level as indicated

The expression of Ki67 was evaluated in the metastatic livers by means of a semi-quantitative scoring procedure (Fig. 5). Cell proliferation was determined to be significantly higher in liver metastases following PH when compared to sham-OP (mean score points 180 vs. 149, *p* < 0.01).

Analysis of proliferation in the surrounding hepatic tissue revealed moderate scores in both groups (PH and sham-OP) for the two investigated regions of interest (invasion zone = 26 vs. 25 score points and area distant to the metastases = 50 vs. 42 score points). No statistical significance ensued from the proliferation scores. However, these scores were well comparable with the growth activity of regenerating healthy livers (32 score points) found 24 h after PH (considered as the “gold standard” when measuring hepatic regenerative response).

## Discussion

PH is considered as a favored treatment option for selected patients with CRC liver metastasis. Even so, liver surgery promotes hepatocytes into multiplication, leading to the reconstitution of lost liver mass. However, this also results in the release of numerous cytokines and growth factors as well as the subsequent activation of multiple pathways, all of which potentially contributing to the growth of residual intrahepatic tumor cells or relatively cryptic satellite metastases and disseminated circulating tumor cells [16]. Liver regeneration is a complex and well-orchestrated process, during which molecular signals may exert dramatic as well as irreversible ‘side effects’ on tumor cells stimulated into proliferation. We employed a rat model of CRC liver metastases to investigate these mutual effects of liver regeneration on the liver itself as well as on the growth of implanted tumor cells.

The syngeneic colon carcinoma cell line CC531, originally derived from a 1,2-dimethylhydrazine-induced rat colon adenocarcinoma, exhibits a reproducible growth pattern and forms ever-increasing tumor masses following intraportal injection into syngeneic WAG/Rij rats. Although this model bypasses the natural evolution of colon cancer, it produces macroscopic liver metastases within 2 weeks after injection of CC531 in up to 85–90 % of animals and is therefore a suitable model to study tumor biology as well as the influence of hepatic regeneration on tumor growth. We noted that the metastatic burden in our rat model displayed a slight preference for the right liver lobules following the chosen direct implantation route via the portal vein. This discovery is interestingly in agreement with findings in human liver, of which the right lobule (Couinaud segments V–VIII) has proven to be the most frequent site (63 % or relatively 67 %) of CRC liver metastasis [17, 18].

### Constitutional expression of cell surface markers and activation of tumor signaling pathways in liver metastases from CC531

To investigate characteristic CRC features, an experimental model mimicking the situation observed in humans is mandatory. We therefore investigated the expression of specific cell surface markers such as CD44 and CD49f in the rat liver metastases. High levels of CD44 expression were demonstrated in the tumors deriving from CC531. This trans-membrane glycoprotein is known to be involved in tumor formation and invasion, cell adhesion and cell migration and it is typically over-expressed in colorectal tumors as opposed to normal mucosa [19]. CD44 is also attributed to distinct ‘stemness’ features in CRC with additional properties such as high mitotic capability, colony formation and drug resistance to conventional chemotherapies [20, 21]. In our study, we also observed rapid tumor growth following implantation of CC531, suggesting that such extensive expression of CD44 in vivo is one feature demonstrating the ‘aggressive’ phenotype of this cell line. However, we have to consider other factors and molecules that could be also involved in the aggressive behavior. As almost all tumor cells are immunoreactive to CD44, we chose this marker as a suitable surrogate parameter to track tumor cells microscopically and to investigate tumor burden on the molecular level (transcripts). Another typical cell surface marker (CD49f = α6 integrin) of CRC was only expressed in some ductular subclones of the rat liver metastases. Integrins play a pivotal role in normal and diseased intestine, as they are responsible for the mediation of cell–matrix interactions such as the specific ligation of various macromolecules (laminins, fibronectins and tenascins) [22]. For example, integrin α5/β1 expression is frequently lost in CRC cells when compared with normal intestinal epithelium; colon cancer cells lacking this integrin are renowned for their extraordinarily proliferative capacity and tumorigenicity [23]. Here in our studies, liver metastases from CC531 were negative for CD49f to an extent of 30 %, suggesting this as a criterion for a profound metastatic cancer cell line.

We also investigated parameters of two representative tumor signaling pathways (wnt and JAK/STAT), which are known to be altered in human CRC. Wnt-Signaling plays an important role in the formation of CRC and efforts to explore therapies designed to target this pathway are in the focus of many research groups [24]. In our study, forming liver metastases from CC531 demonstrated membrane-bound expression of the frizzled-receptor as well as intense cytoplasmatic expression of β-catenin. Both markers may represent the constitutive activation of the wnt-pathway in our rat model. Furthermore, STAT3 is aberrantly activated in human CRC tissue and found to correlate to malignant tumor progression through the up-regulated expression of matrix metalloproteinases [25, 26]. In our study, we also demonstrated high nuclear expression in liver metastases derived from CC531.

In summary, we demonstrate that our experimental model is not only feasible and reproducible, but also mimics well observations in humans and could therefore be considered as a suitable pre-clinical rat animal model for the further study of the behavior of orthotopic CRC liver metastases following the stimulus of PH.

### Proliferation of liver metastases and effects on the surrounding liver tissue following PH

The hepatic regeneration process is triggered promptly after injury. In rats, DNA replication begins as early as 16 h after PH [27]. Between 5 and 7 days, the liver volume is almost completely restored (approx. 98 %) through hyperplasia of the remaining lobes [16]. However, even after complete recovery of the total organ mass, the regeneration and remodeling process may continue. At the endpoint in our studies (2 weeks following injection of CC531), we observed ongoing DNA synthesis, as illustrated by the marked immunoreactivity to Ki67 in liver metastases in both groups, significantly more so in the PH group on direct comparison. It is worth noting that substantially elevated Ki67 scores were also detected in the surrounding liver parenchyma, which may also suggest continued cell proliferation through presumably paracrine or space-requiring effects as a result of the stimulated expansion of metastatic tissue towards the end or even after termination of liver regeneration (10–14 days following PH). In accordance, Harun et al. [28] also detected long-lasting and significantly greater numbers of proliferating tumor cells as a function of the extent of liver resection as late as on day 21 following PH (37 and 70 %).

Histological analysis revealed a ‘pushing’ growth pattern of rat CRC liver metastases. They certainly appear to exert a relevant effect on the surrounding liver plate, leading to this characteristic re-arrangement of adjacent hepatocytes. Of note, this histological pattern is most frequent (approx. 35 %) in human CRC liver metastases and was found to be an independent predictor of poor survival (at 2 years follow-up) suggesting the more aggressive tumor biology behind this phenomenon [15]. Cx32 has been studied to investigate the ‘gap function’ and maintenance of tissue homeostasis in the liver and it plays an important role in monitoring the loss of functionality during the course of liver disease [29]. In our studies, we also observed a loss of this hepatic marker in the tumor-liver parenchyma interface of 2–4 hepatocyte layers in direct proximity to the liver metastases. In accordance to the observed ‘pushing’ growth pattern, this impairment of cell integrity in hepatocytes (‘pushed’ liver margin) underlines the profound impact exerted by tumor cell growth on the surrounding liver tissue.

### Evaluation strategies and PH as a significant impact on tumor growth

Several studies have already investigated the stimulatory effects of PH on liver metastases in the past [28, 30–34]. Most commonly, wet liver weight, macroscopic assessment or histological analysis methods were used to measure the hepatic tumor burden with obvious limitations for the exact evaluation of the morphometric data. In our study, we employed MRI T2-weighted images to evaluate tumor growth in all liver lobules as well as in extrahepatic sites. Over nearly a decade, MRI has emerged as the gold standard imaging technique in the detection and characterization of liver lesions owing to its high specificity resulting from optimal lesion-to-liver contrast and lack of ionizing radiation [35]. The implementation of this non-invasive, high-resolution imaging modality enabled us to calculate and quantify the extent of the hepatic tumor burden in our pre-clinical rat model to a much greater degree of accuracy. In more detail, we demonstrated that 1/3 PH was well associated with a volumetric increase by a factor of nearly 3 (2.8-fold) in the RTM. Our MRI results were confirmed on the molecular level, as we found similar results, such as a 2.3-fold amplification in gene expression of CD44—the surrogate marker for tumor burden in our studies.

We believe it is important to reiterate at this point that PH was performed 24 h following implantation of CC531. Thus, anything up to 30 % of the CC531 cells were removed through PH. As a direct result of this technically unavoidable loss, the initial tumor cell load was always lower in the PH group. Even so, we still detected a significant increase in tumor burden after 2 weeks. Since we were unable to determine numbers of CC531 cells lost to PH, this was not taken into account when comparing and contrasting the PH group with the sham-OP group.

We would like to add that we specifically used an immunocompetent animal model with syngeneic and orthotopic implantation of CRC tumor cells to produce liver metastases, aiming to be as close to the clinical situation as possible and taking into account the very important role of tumor cell growth in the presence of an intact immune system [36]. Many (if not most) other animal models use heterotopic implantation sites (e.g. limbs) to simplify the process of tracking the fate of tumor cell growth. These methods often employ direct sight and/or vector imaging technologies based on the insertion of a reporter gene (e.g. red fluorescent dye) into the cancer cell lines, emitting in the near-infrared spectrum for non-invasive, high-resolution and life-time detection in the tissue [37, 38]. However, these fluorescence-labeling gene delivery systems are predominantly used in nude (immunodeficient) mice. The insertion of a fluorescent protein tag is immensely capable of activating the host immune system, leading to the complete elimination of engrafted labeled tumor cells (own observation following stable transfection of the red fluorescent marker mCherry into CC531). In contrast, we were able to perform native MRI scans without any need to employ potentially immunogenic or harmful substances (proteins, antibodies, viral vectors) to mediate the imaging.

When considering the effects of PH on the growth of liver metastases in animal models, a number of studies have determined that the extent of hepatic resection is associated with a higher incidence and volume of tumor recurrence [39, 40]. The direct correlation between PH and the stimulatory effect on tumor growth was also demonstrated by Panis et al. [41, 42]. Colon carcinoma cells (DHD K12) were injected into the portal vein of BD IX rats and only 40 % of animals had developed liver metastases within 8 weeks. However, when 2/3 PH was performed, the incidence of liver metastases increased to 62 %. It appears most likely that liver micrometastases were present, but did not begin development until stimulated by liver regeneration. Slooter et al. [31] revealed that PH significantly increased the number of liver metastases following PH and successfully used a concept of adjuvant treatment with TNF-α to reduce the number of outgrowing metastases. However, Harun et al. [28] could only confirm a significant stimulatory effect on tumor growth if the mice underwent a large 2/3 PH and the CRC cells were implanted at a relatively late stage in liver regeneration (on day 6 post PH). In comparison, only a very few studies have shown that minor resection of less than 50 % did not result in tumor stimulation [43, 44]. In contrast, our own work demonstrates that 1/3 PH is more than able to exert a proliferative effect on CRC liver metastases, suggesting that there is indeed no clear threshold below which tumor cells are no longer stimulated. However, tumor cell engraftment and the outgrowth of metastases may very much depend on the number of cells implanted as well as the type of cancer cell line as basis.

The differences in liver volume increase and metastatic burden following PH or sham-OP was clearly demonstrated by MRI. However, since we used clinical MRI hardware and conventional MRI contrast, we may have underestimated the entire tumor burden by missing out small metastases and satellite tumors in the liver parenchyma due to the limited resolution (1 mm slices of 0.33 mm). We will therefore employ gadolinium-based contrast agents in our future MRI studies, as this change in protocol may further enhance the demarcation of the tumor masses in the liver [45, 46]. This change may be key in the assessment of limited tumor growth, especially at earlier points in time following implantation, either during the initial phase (1–3 days following PH) or the ongoing course of liver regeneration (within 5–7 days). Of note, we will employ the gadolinium-based contrast agent gadobutrol (Gadovist/Gadavist^®^, Bayer Pharma AG, Berlin, Germany) in a non-toxic chelated preparation which exhibits negligible protein binding, distributes in extracellular space only, and is rapidly excreted via the renal pathway. It is widely used in humans to detect hepatic lesions or liver metastases respectively and well tolerated following intravenous injection either of a diagnostic single or repeated doses, with no relevant toxicity to the liver in either humans or rodents [47–49].

### Evaluation of molecular changes in CRC liver metastases following PH

We investigated the molecular features in homogenates from tumor-bearing livers, presuming that the transcript levels of the molecular markers Axin2, CXCR4, CD49f and c-met were predominantly attributed to the metastatic burden, as they were barely detectable in normal livers or in livers from control animals. We considered a panel of markers for the investigation of specific tumor characteristics, aspects of invasiveness and aggressiveness, all known to promote tumor cell growth in the liver parenchyma: Axin2 is well known as an important target gene of the canonical wnt-signaling cascade constitutively activated in CRC [50]. The chemokine receptor CXCR4 contributes to tumor growth and the metastatic spread of several cancer entities [51]. CD49f, as mentioned above, was chosen as an essential ligand for tumor cell adhesion. Hepatocyte growth factor (HGF) is a key player during the proliferation phase of liver regeneration and synergistically acts in tumor progression. Its high-affinity receptor c-met is frequently amplified or over-expressed in CRC and high expression levels are associated with cancer progression, metastatic growth and invasiveness [52]. Surprisingly, we were unable to detect any changes in the relative mRNA expression levels of the molecular markers in metastatic liver following PH. This may suggest that the stimulus derived from liver regeneration acted on the CC531 cells in a uniform fashion to increase the metastatic mass, but did not result in any preferential stimulation of individual subclones.

### Conclusion and therapeutic implications

Liver regeneration is arguably the most fascinating and complex regenerative response to injury. It follows an intricate network of cytokines and cell-growth factors mediating the restoration of liver function and morphology [53]. However, there is mounting clinical evidence that liver regeneration can be both beneficial and detrimental, depending on the nature of the cell responding to the proliferation stimulus [54].

It is important to recognize the limitations of our rat model of CRC liver metastasis as a pre-clinical research environment. Firstly, this animal model will never approach the complexity of the human situation of continuing tumor development and formation of metastasis. Secondly, PH was actually performed in active tumor disease (liver resection 24 h following tumor cell implantation). However, the present study was specifically designed to identify the extent of 1/3 PH as a stimulatory effect on the growth of CRC rat liver metastases and on their molecular composition. Both issues were examined in this rat animal model with clear results. Furthermore, our present work may serve as a pilot study for more detailed investigations addressing the fundamental questions as to whether the molecular and cellular mechanisms of tumor cell growth are related to the three different phases of liver regeneration (1: priming (ECM degradation), 2: proliferation (DNA synthesis) and 3: termination (ECM remodeling and angiogenesis)). It has been suggested that molecules regulating the late phase of liver regeneration may be the key factors in tumor cell growth and disease progression [54]. Thus, future studies need to determine possible targets and therapeutic applications as to how to hinder the natural liver regeneration response at least in part in such a manner without any acute detriment, in order to effect the necessary regenerative response of a liver already impaired in a likely clinical setting of multimodal treatment concepts following neo-adjuvant chemotherapy and/or post-operative adjuvant systemic therapy [55].

It is worth mentioning that de Jong et al. [56, 57] provided a proof of principle that radioimmunotherapy using a radiolabeled monoclonal antibody was effective in the treatment of at least microscopic liver metastases and was additionally effective in an adjuvant treatment modality after surgery. When referring to larger CRC metastases, cell-cycle-blocking agents such as ionizing radiation could be considered not only to eliminate the liver metastases themselves, but also to reduce the regenerative response of the surrounding liver mass as a method of pre-treatment prior to liver resection. For example, external-beam and partial liver irradiation may prove promising in this difficult multidisciplinary approach, as the dose of radiation can be adjusted and precisely targeted to parts of the liver [55, 58]. No doubt, future studies will be necessary to clarify the underlying mechanisms, treatment options and understanding of CRC metastatic recurrence in regenerating liver.
